# The Importance of Nutritional Aspects in the Assessment of Inflammation and Intestinal Barrier in Patients with Inflammatory Bowel Disease

**DOI:** 10.3390/nu14214622

**Published:** 2022-11-02

**Authors:** Olga Kaczmarczyk, Agnieszka Dąbek-Drobny, Agnieszka Piątek-Guziewicz, Michał Woźniakiewicz, Paweł Paśko, Justyna Dobrowolska-Iwanek, Aneta Woźniakiewicz, Aneta Targosz, Agata Ptak-Belowska, Urszula Szczyrk, Małgorzata Strzałka, Paweł Zagrodzki, Małgorzata Zwolińska-Wcisło

**Affiliations:** 1 Department of Gastroenterology and Hepatology, Jagiellonian University Medical College, 31-008 Krakow, Poland; 2 Unit of Clinical Dietetics, Department of Gastroenterology and Hepatology, Jagiellonian University Medical College, 31-008 Krakow, Poland; 3 Department of Analytical Chemistry, Faculty of Chemistry, Jagiellonian University, Gronostajowa 2, 30-387 Krakow, Poland; 4 Department of Food Chemistry and Nutrition, Jagiellonian University Medical College, 31-008 Krakow, Poland; 5 Department of Physiology, Faculty of Medicine, Jagiellonian University Medical College, 31-531 Krakow, Poland

**Keywords:** intestinal inflammation, nutrition, dietary fiber, SCFA, tight junction proteins, TNF-α, PPAR-γ, succinate, iNOS

## Abstract

Intestinal inflammation in inflammatory bowel disease (IBD) is closely linked to nutrition. This study aimed to evaluate associations between nutritional, inflammatory, and intestinal barrier parameters in patients with IBD. We assessed nutritional status, fecal short-chain fatty acid profile, serum cytokine levels, and mRNA expression of enzymes and tight junction proteins in intestinal biopsies obtained from 35 patients, including 11 patients with inactive IBD, 18 patients with active IBD, and six controls. Patients with active IBD were characterized by hypoalbuminemia, fluctuations in body weight, and restriction of fiber-containing foods. In addition, they had significantly reduced levels of isovaleric acid and tended to have lower levels of butyric, acetic, and propionic acids. Patients with active IBD had higher mRNA expression of peroxisome proliferator-activated receptor γ and inducible nitric oxide synthase, and lower mRNA expression of claudin-2 and zonula occludens-1, compared with patients with inactive IBD. Moreover, patients with a body mass index (BMI) of ≥25 kg/m^2^ had higher median tumor necrosis factor-α levels that those with a lower BMI. We comprehensively evaluated inflammatory parameters in relation to IBD activity and nutritional status. The discrepancies between proinflammatory and anti-inflammatory parameters depending on IBD activity may be related to nutritional factors, including diet and abnormal body weight.

## 1. Introduction

Inflammatory bowel disease (IBD) is a term for two main conditions, Crohn’s disease (CD) and ulcerative colitis (UC), characterized by relapsing intestinal inflammation and a largely unknown etiology. Currently, the etiopathogenesis of IBD is understood as the complex interaction between genetic, environmental, microbiological, and immune factors [1]. Several proinflammatory cytokines are known to be involved in the pathogenesis of IBD, including tumor necrosis factor α (TNF-α) as well as interleukins IL-6 and IL-17, and modifying their production has shown benefits for disease control [2,3]. A growing body of evidence points to an important role of intestinal barrier disruption in the pathogenesis of IBD [4]. This barrier consists of a layer of epithelial cells that are tightly connected by intercellular junctions, such as tight junctions (TJs), adherens junctions, and desmosomes. A recent study showed that IBD is associated with altered TJ protein expression, including transmembrane proteins, such as occludins, claudins, and scaffolding proteins zonula occludens [5,6]. These changes result in increased paracellular permeability and antigen influx as well as exacerbation of intestinal inflammation and clinical symptoms, such as diarrhea, abdominal cramps, and bloating [2,4].

Currently, increasing attention is being paid to nutritional factors that can modify the course of intestinal inflammation. One of them is the intake of dietary fiber, which affects the production of beneficial short-chain fatty acids (SCFAs), such as acetate, propionate, and butyrate. These metabolites of bacterial fermentation of fiber strengthen barrier integrity and reduce inflammatory response by regulating the nuclear factor kappa B (NF-κB) signaling pathway and cytokine production [7]. Recent studies showed that SCFAs, especially butyrate, regulate the expression of TJ proteins [8]. In addition, SCFAs induce an anti-inflammatory response by modifying the cytokine release and expression of intestinal enzymes, such as inducible nitric oxide synthase (iNOS) and peroxisome proliferator-activated receptor γ (PPAR-γ), which are important for immune response regulation [9].

Despite the beneficial properties of fiber metabolites, numerous patients with IBD modify their diets, limiting their fiber sources for fear of symptom exacerbation [10]. This may translate into changes in the composition of intestinal microbiota and SCFAs, worsening of inflammation, and disturbances in intestinal barrier function [11,12]. However, not all dietary fiber metabolites have a proven positive effect on intestinal homeostasis. One of them, succinate, may act as an active proinflammatory mediator that promotes intestinal fibrosis. However, its exact role in intestinal inflammation remains unknown [13]. Nevertheless, it seems that an adequate supply of dietary fiber, together with a balanced gut microbiota, may reduce inflammation and strengthen the intestinal barrier in patients with IBD.

Nutritional aspects, such as malnutrition, reduced oral intake, and weight fluctuations, are other important factors that may affect the course of IBD [14]. Malnutrition is associated with exacerbation of inflammation and may lead to the need to modify treatment, which consequently also affects the intestinal barrier. It should be noted that malnutrition affects not only underweight patients, but also those with normal and excessive body weight [15]. Increasing attention is now being paid to the role of excessive body weight in ongoing inflammation, as adipose tissue is both immunologically and metabolically active [16]. This is especially important for patients with IBD, as the percentage of overweight and obese patients in this population is increasing [17].

Given the interaction of multiple factors in IBD, the aim of the present study was to comprehensively evaluate the parameters of inflammation and the intestinal barrier, as well as the SCFA profile, with consideration of nutritional factors such as fiber intake, malnutrition, and abnormal body weight. For this purpose, we aimed to evaluate blood inflammatory parameters, such as IL-6, IL-10, IL-17, IL-22, and TNF-α, and intestinal mRNA expression of iNOS, PPAR-γ, hypoxia-inducible factor (HIF-1α), zonula occludens-1 (ZO-1), claudin-2, occludin, and succinate receptor 1 (SUCNR1) in IBD patients depending on disease activity. Finally, we attempted to determine the most important associations between these factors.

## 2. Materials and Methods

### 2.1. Study Population

The study population included patients with IBD diagnosed on the basis of clinical, endoscopic, histological, and radiological examinations in accordance with the European Crohn’s and Colitis Organization’s consensus guidelines [18]. Colonic involvement was required in patients with CD and at least left-sided colitis in patients with UC. Patients were divided into groups with inactive and active disease according to the full Mayo criteria for UC and the Crohn’s disease activity index and simple endoscopic score for Crohn’s disease for CD. All participants completed a nutritional questionnaire, which was an abbreviated version of the food frequency questionnaire. Blood samples were collected for standard blood tests and cytokine measurement, while fecal samples were collected for calprotectin and SCFA determination. Moreover, a colonoscopy or sigmoidoscopy was performed to evaluate the severity and extent of inflammatory lesions in the large intestine, to obtain biopsies for histopathological examination, and to assess the mRNA expression of selected parameters.

Controls included volunteers with functional bowel disorders, without organic and inflammatory lesions in the large intestine in a colonoscopy, and who did not meet Rome IV diagnostic criteria for irritable bowel syndrome. The exclusion criteria were as follows: morbid obesity (body mass index [BMI] > 40 kg/m^2^), pregnancy, lactation, acute infections, malignancies, alcohol abuse, smoking, eating and metabolic disorders, serious mental illness, celiac disease, biologic therapy, use of prebiotics and probiotics, including supplements containing SCFAs, partial and total parenteral nutrition, and severe somatic disorders not related to IBD.

All participants provided their written informed consent to participate in this study. The study was conducted in accordance with the Declaration of Helsinki and was approved by the Bioethics Committee at Jagiellonian University in Krakow, Poland (no. 1072.6120.18.2018; as of 23 February 2018).

### 2.2. Nutritional Questionnaire

The nutritional questionnaire concerned eating habits in the last 3 months and was a shorter version of the food frequency questionnaire. All participants received instructions before completing the questionnaire. In our study, the original version was limited to questions regarding the frequency of consumption of selected product groups: gluten products, fresh vegetables and fruits, dried fruits, wholegrain products, legumes, and dairy products. Moreover, the frequency of fiber consumption was assessed according to responses provided by participants. Participants were assigned to one of the 2 groups: low-fiber (<20 g/day) and normal-fiber (>20 g/day) consumption, depending on the type of diet used.

### 2.3. Biochemical Analysis of Selected Parameters in Serum and Fecal Samples

Biochemical tests, including the measurement of complete blood count, C-reactive protein (CRP), albumin, and calprotectin levels, were performed at the Department of Diagnostics of the University Hospital in Krakow, Poland, in accordance with relevant laboratory procedures.

Serum cytokine (IL-6, IL-10, IL-17, IL-22, and TNF-α) and zonulin levels were determined at the Department of Physiology, Laboratory of Genetics and Molecular Biology, Faculty of Medicine, Jagiellonian University Medical College, using commercially available enzyme-linked immunosorbent assay kits according to the manufacturer’s protocol. The detection kits for IL-10, IL-17, IL-22, and zonulin were purchased from Shanghai Sunred Biological Technology Co. (Shanghai, China), while those for IL-6 and TNF-α were purchased from LDN Labor Diagnostika Nord GmbH and Co. KG (Am Eichenhain, Berlin, Germany). These included Human IL-10 catalog number 201-12-0103D, Human IL-17 catalog number 201-12-0143, Human IL-22 catalog number 201-12-0039, Human Zonulin catalog number 201-12-5578, IL-6 ELISA catalog number IL E-3200, and TNF-α ELISA catalog number IL E-3100. An ELx 808 spectrophotometric microplate reader (BioTek, Winooski, VT, USA) was used to determine the optical density at 450 nm.

### 2.4. Determination of Organic Acid Levels in Fecal Samples

All fecal samples obtained were immediately frozen at −80 °C until further analysis. Stool samples were prepared and extracted at the Department of Food Chemistry and Nutrition, Faculty of Pharmacy, Jagiellonian University Medical College, using the methods described in our previous work [19,20]. The process involved successive drying, grinding, and then triple extraction.

The levels of acetic, lactic, propionic, butyric, isobutyric, valeric, isovaleric, and succinic acids were assessed using capillary electrophoresis with ultraviolet spectrophotometric detection, PA 800 plus pharmaceutical analysis system (Beckman Coulter, Brea, CA, USA) at the Laboratory for Forensic Chemistry, Faculty of Chemistry, Jagiellonian University. Samples were injected hydrodynamically (3.45 kPa for 8 s). The separation process was conducted in a fused silica capillary (60 cm in total length, with an internal diameter of 75 µm) at 25 °C with a high-voltage of −30 kV applied. The spectrophotometric detection was performed at 230 nm using an indirect detection mode.

### 2.5. Evaluation of Intestinal Biopsies for mRNA Expression of Selected Parameters Using Real-Time Polymerase Chain Reaction

All endoscopies with intestinal biopsies were performed at the Department of Gastroenterology and Hepatology of the University Hospital in Krakow. In patients with inactive IBD, biopsy samples were obtained from previously affected intestinal mucosa, and in patients with active IBD samples were obtained from inflamed intestinal mucosa. In the control group, a sample of the healthy colonic mucosa was obtained. Intestinal biopsies were frozen in liquid nitrogen immediately after collection and stored at −80 °C for further analysis.mRNA expression of iNOS, PPAR-γ, HIF-1α, ZO-1, claudin-2, occludin, SUCNR1, IL-6, and β-actin as reference gene was determined in intestinal biopsies by real-time polymerase chain reaction (PCR). Total RNA was isolated from biopsies using a commercially available kit with spin-columns (ReliaPrep™ RNA Miniprep Systems, Promega Corporation, Madison, WI, USA) according to the manufacturer’s protocol. The RNA concentration was measured using Nanodrop ND-1000 (ThermoFisher, Waltham, MA, USA). Reversed transcription to cDNA was performed using Reverse Transcription System A3500 (Promega Corporation). Reversed transcription was normalized for each reaction regarding the total RNA concentration to obtain the same value (1 μg) for each sample. mRNA expression for iNOS, PPAR-γ, HIF-1α, ZO-1, claudin-2, occludin, SUCNR1, IL-6, and β-actin was determined using specific primers. β-Actin as reference gene was determined using 5′-CACCATTGGCAATGAGCGGTTC-3′ forward and 5′-AGGTCTTTGCGGATGTCCACGT-3′ reverse primers; iNOS was determined using 5′-GCTCTACACCTCCAATGTGACC-3′ forward and 5′-CTGCCGAGATTTGAGCCTCATG-3′ reverse primers; PPAR-γ was determined using 5′-AGCCTGCGAAAGCCTTTTGGTG-3′ forward and 5′-GGCTTCACATTCAGCAAACCTGG-3′ reverse primers; HIF-1α was determined using 5′-TATGAGCCAGAAGAACTTTTAGGC-3′ forward and 5′-CACCTCTTTTGGCAAGCATCCTG-3′ reverse primers; ZO-1 was determined using 5′-GCCGCTAAGAGCACAGCAA-3′ forward and 5′-TCCCCACTCTGAAAATGAGGA-3′ reverse primers; claudin-2 was determined using 5′- GTGACAGCAGTTGGCTTCTCCA-3′ forward and 5′-GGAGATTGCACTGGATGTCACC-3′ reverse primers; occludin was determined using 5′-ATGGCAAAGTGAATGACAAGCGG-3′ forward and 5′-CTGTAACGAGGCTGCCTGAAGT-3′ reverse primers; SUCNR1 was determined using 5′-CTGCTCTGCCCCTTGAAAAG-3′ forward and 5′-TGACGACCTGAGTGCACTGATAC -3′ reverse primers; and IL-6 was determined using 5′-AGACAGCCACTCACCTCTTCAG-3′ forward and 5′-TTCTGCCAGTGCCTCTTTGCTG-3′ reverse primers. All primers were synthesized by Sigma-Aldrich (Sigma Aldrich, St. Louis, MO, USA). The real-time PCR was conducted using a Rotor Gene RG-3000 thermal cycler (Corbett Research Ltd., Saffron Walden, UK) and GoTaq^®^ qPCR Master Mix (Promega Corporation, Madison, WI, USA). The same amount of cDNA per well was used to maintain the same PCR reaction efficiency in all analyzed samples. After the reaction, the melting curve for each sample, its technical replicates, and appropriate negative control were evaluated to exclude data derived from potentially unintended products. Results were examined using the −ΔΔCt method [21].

### 2.6. Statistical Analysis

The differences between groups were assessed using the analysis of variance with the post-hoc Tukey test (for parameters with normal distribution and homogenous variances). The Kruskal–Wallis test with a post-hoc Dunn test and the Mann–Whitney test were used for parameters with nonnormal distribution. A probability level of less than 0.05 was considered significant.

Statistical analyses were carried out using Graph Pad Prism v.3.02 (GraphPad Software, San Diego, CA, USA) and the STATISTICA v. 13.3. package (TIBCO Software Inc., Palo Alto, CA, USA).

## 3. Results

### 3.1. Characteristics of Study Participants

A total of 35 patients were enrolled in the study, including seven patients with CD, 22 patients with UC, and six controls. The characteristics of participants and the results of standard tests are shown in Table 1. There were significant differences in the levels of inflammatory markers such as CRP and calprotectin between study groups (Table 1). In addition, patients with active IBD had lower mean albumin levels than patients with inactive IBD and controls, but the results were not significant. The mean BMI was higher in patients with active IBD than in those with inactive IBD, but the difference was again nonsignificant. The clinical manifestations of patients with IBD are presented in Table 2.

### 3.2. Nutritional Status

In the active IBD group, eight patients (44.4%) reported a low-fiber diet and nine patients (50%) reported dairy product consumption. In the inactive IBD group, nine patients (81.9%) followed a low-fiber diet and six participants (54.5%) regularly consumed dairy products. As for the body weight, 61% of patients with active IBD reported fluctuations in body mass in the previous six months, compared with 54% of patients with inactive IBD. The frequency of consumption of selected food groups is presented in detail in Table 3, Table 4 and Table 5.

### 3.3. Fecal Organic Acid Concentrations

The median levels of organic acids in study groups are presented in Table 6. The median isovaleric acid level was significantly lower in patients with active IBD than in patients with inactive IBD and controls (Table 6). In addition, butyric, propionic, and acetic acid levels were lower in patients with active IBD compared with those with inactive IBD, but the difference was not significant. Patients with inactive IBD had the highest median levels of succinic acid, but again the difference was not significant (Table 6). Moreover, patients on a low-fiber diet had higher median succinic acid levels than those on a diet with a normal amount of fiber (527.4 µg/g vs. 178.14 µg/g; *p* < 0.05; Figure S1). On the other hand, the median lactic acid level was higher (not significantly) in patients with active IBD than in those with inactive IBD or controls, and it was higher in patients on a diet with a normal amount of fiber compared with those on a low-fiber diet (1146.71 µg/g vs. 434.13 µg/g; *p* < 0.05; Figure S2). Moreover, the median butyric acid level was lower in patients with hypoalbuminemia compared with those with normal albumin levels (below the limit of determination [LOD] vs. 220.87 µg/g; *p* < 0.05). Significant differences in the SCFA profile were also shown for patients with diarrhea. They had higher median lactic acid levels and lower median propionic and isovaleric acid levels, compared with patients with normal bowel movements (Figure S3).

### 3.4. Serum Cytokine and Zonulin Concentrations

Serum cytokine and zonulin levels are demonstrated in Table 7. Patients with IBD had higher (not significantly) median TNF-α levels compared with controls. Moreover, median TNF-α levels were higher in patients with a BMI of ≥25 kg/m^2^ compared with those with a BMI of <25 kg/m^2^ (14.13 pg/mL vs. 3.04 pg/mL; *p* < 0.05; Figure S4). The highest median zonulin levels were noted in patients with inactive IBD (Table 7), but there were no significant differences in zonulin levels according to BMI. No correlation was observed between blood cytokine and zonulin levels and dietary fiber intake or albumin levels.

### 3.5. The mRNA Expression of iNOS, PPAR-γ, HIF-1α, ZO-1, Claudin-2, Occludin, SUCNR1, and IL-6 in Intestinal Biopsies

**Figure 1:**
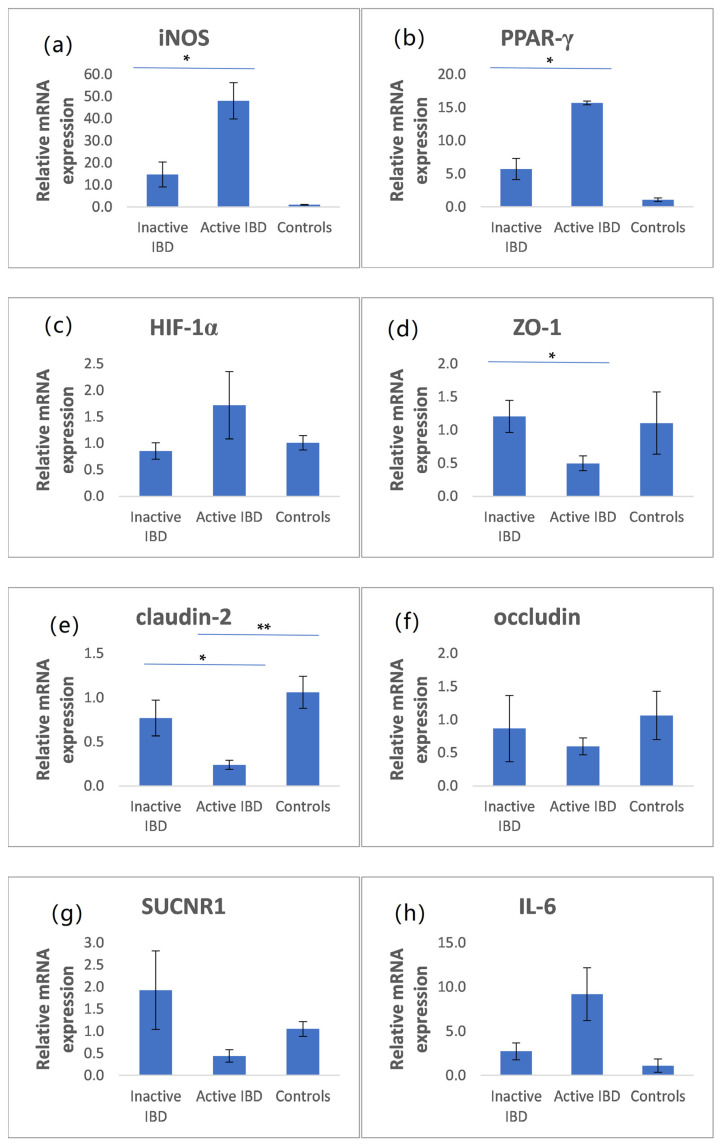
Relative mRNA expression of inducible nitric oxide synthase (iNOS), peroxisome proliferator-activated receptor γ (PPAR-γ), hypoxia-inducible factor 1α (HIF-1α), zonula occludens-1 (ZO-1), claudin-2, occludin, SUCNR1, and interleukin IL-6 (**a**–**h**) in colonic tissue of patients with IBD, active IBD, and controls (mean ± SE). * *p* < 0.05 ** *p* < 0.01.

The mRNA expression of iNOS, PPAR-γ, HIF-1α, ZO-1, claudin-2, occludin, SUCNR1, and IL-6 is presented in Figure 1. Patients with active IBD had significantly higher iNOS and PPAR-γ mRNA expression than those with inactive IBD (Figure 1a,b). In addition, claudin-2 and ZO-1 mRNA expression was significantly lower in patients with active IBD than in those with inactive IBD (Figure 1d,e). The mRNA expression of HIF-1α and IL-6 was higher in active IBD compared with inactive IBD and controls, but without statistical significance. Similarly, the mRNA expression of occluding and SUCNR1 was lower in active IBD in comparison with controls and inactive IBD, but was also not statistically significant (Figure 1c,f–h).

### 3.6. Investigation of Possible Gender Bias for the Studied Population

In the case of the largest group of subjects (active IBD), the influence of gender on the values of the parameters tested was checked. With the exception of BMI (medians: 19.2 kg/m^2^ vs. 23.6 kg/m^2^, *p* = 0.032, for females and males, respectively), no gender effect was found for all other parameters (*p* within the range 0.1247–1.000).

## 4. Discussion

In this study, we comprehensively evaluated inflammatory and intestinal barrier parameters depending on disease activity and nutritional status in patients with IBD. We demonstrated that these parameters should be assessed together with nutritional aspects, such as BMI, albumin levels, and fluctuations in body weight.

This study showed that patients with active IBD are characterized by elevated levels of standard inflammatory markers (including CRP and calprotectin), reduced albumin levels, and a higher prevalence of fluctuations in body weight, including both weight loss and excess weight gain, compared with patients with inactive IBD. In addition, almost 80% of patients with active disease do not consume wholegrain products, and more than 60% do not eat dried fruits and legumes. Previous studies revealed that patients with IBD report numerous food products that they believe have worsened their symptoms and modify their diets despite the lack of strong scientific evidence for such a link [10]. This can lead not only to malnutrition and micronutrient deficiencies but also to a reduction in the number of substrates needed to produce beneficial SCFAs [22]. Our study showed a trend toward lower levels of the main SCFAs such as butyric, propionic, acetic, and isovaleric acids in patients with active IBD. This may be caused both by a reduced consumption of fiber-rich foods, which we noted among our participants, and by the disruption of the gut microbiota reflected in reduced amounts of SCFA-producing bacteria [23].

In our study, more than 30% of IBD patients were overweight or obese. Our findings are consistent with recent research reporting that the proportion of overweight IBD patients increased by 20–40% and those with obesity increased by 15–40% [17]. This leads to overnutrition, resulting in worse clinical outcomes such as increased rates of complications and hospitalizations [17]. Excessive body weight with visceral adipocyte hypertrophy leads to an increased production of proinflammatory cytokines, including TNF-α, IL-1, and IL-6, and aggravation of intestinal inflammation [17,24]. Moreover, obesity appears to be associated with frequent colonic involvement in patients with CD [17]. This is in line with our observations that showed the highest median TNF-α level in patients with BMI ≥ 25 kg/m^2^ compared with patients with lower BMI, as well as mean BMI, was higher in active IBD in comparison with inactive IBD, but without statistical significance. These results seem to support the fact that adipose tissue, in addition to other functions, is an immune organ significantly contributing to inflammatory processes. Of note, another risk factor for weight gain is corticosteroid therapy used to induce remission in IBD patients. In our study, over 70% of patients used corticosteroids in the previous year. Recent research demonstrated that approximately 24% of patients taking corticosteroids for more than one year gained weight of more than 10 kg [17].

The analysis of intestinal barrier parameters revealed higher iNOS mRNA expression in patients with active IBD than in those with inactive IBD. This is in line with several previous studies showing that IBD is associated with higher iNOS expression, resulting in an elevated production of cytotoxic nitric oxide [25]. The overexpression of nitric oxide leads to exacerbation of inflammation and more severe diarrhea [25]. We hypothesized that reduced SCFA levels in patients with active IBD may be an important factor contributing to increased iNOS expression, as SCFAs were demonstrated to inhibit iNOS expression by the NF-κB pathway [26,27].

This study found that patients with active IBD had higher intestinal PPAR-γ expression than patients with inactive IBD. This is contrary to the previous reports of PPAR-γ as an anti-inflammatory factor that regulates the multiple pathways of immune response, including the regulation of iNOS expression and activation of the NF-κB, STAT-1, and AP-1 signaling pathways [28,29]. However, PPAR-γ is also an important regulator of glucose and lipid metabolism used in the treatment of metabolic diseases [30]. Studies demonstrated that PPAR-γ expression is affected by several external factors, such as medication use (e.g., mesalazine) and dietary pattern [31]. It was reported that a high-fat diet (including Western diet) and obesity positively regulate the PPAR-γ expression, while fasting and a low-calorie diet have a negative effect [32,33]. In contrast to our results, previous studies revealed reduced PPAR-γ expression in IBD, especially in active disease, which may be related to disease progression [34,35]. Hence, high PPAR-γ expression, which we noted in patients with active IBD, may be due to dietary modification, such as high fat intake and excess body weight. During disease exacerbation, patients with IBD tend to change their daily diet to include wheat products and processed, easily digestible foods to alleviate their symptoms, which is in line with the Western style of nutrition. Another possibility is the induction of the PPAR-γ expression by intestinal microorganisms [33]. As IBD is associated with disruption of the intestinal microbiome (dysbiosis), it is necessary to establish whether the disease is associated with increased amounts of PPAR-γ-activating bacteria, especially in the presence of abnormal body weight. However, despite high PPAR-γ expression in intestinal tissue, our patients also showed clinical and endoscopic evidence of disease exacerbation as well as high iNOS mRNA expression. Our finding suggests that the PPAR-γ-dependent pathway is one of the many signaling pathways in a complex network of inflammatory responses and may not be crucial in the anti-inflammatory response during colitis. However, modification of this pathway may be particularly important in IBD patients with an abnormal body weight.

In this study, we also assessed the expression of TJ proteins, which are key structures for maintaining the proper function and paracellular permeability of the mucosal barrier. We found that patients with active IBD had lower intestinal expression of the following TJ proteins: occludin, claudin-2, and ZO-1. This is in line with previous studies showing reduced occludin and ZO-1 expression in IBD patients, resulting in increased permeability [36,37]. However, in contrast to our findings, recent studies revealed that pore-forming claudin-2 expression was increased in IBD and correlated with the severity of inflammation [37,38]. This discrepancy may be related to the unexpected finding of elevated PPAR-γ mRNA expression in patients with active IBD, because the activation of the PPAR-γ signaling pathway reduces claudin-2 expression [39]. Another possible explanation is the fact that patients with CD and UC differ in the expression of TJ proteins [6]. Moreover, some reports indicate that SCFAs regulate the intestinal barrier by modulating the expression of TJ proteins [40]. It is still uncertain which acid is the most important for barrier stability and what the underlying mechanism is. In our study, we did not show any association between individual acids and TJ proteins, but we cannot exclude the possibility that reduced levels of major SCFAs lower the expression of these proteins in active IBD.

Interestingly, we observed a trend toward higher succinic acid levels in stool as well as increased SUCNR1 mRNA expression in the colon of patients with inactive IBD. Recent studies also reported increased levels of succinate in serum and stool as well as increased intestinal expression of SUCNR1 in IBD patients [13,41,42]. In addition, some authors demonstrated a correlation between disease activity and succinate accumulation in a murine colitis model [43]. Moreover, previous studies revealed that elevated succinate levels are associated with microbial imbalance in IBD [42]. This is consistent with our findings that patients on a low-fiber diet, which alters the diversity and abundance of bacterial species [44], have lower levels of succinic acid. Succinate is a metabolite of dietary fiber that activates SUNCR1 present in several cells, including epithelial and immune cells of the human intestines. So far, the results of studies on the role of succinate accumulation remain conflicting, and it appears that its effect depends on the ongoing pathology and the site of inflammation (local vs. systemic). Some studies have indicated that the accumulation of extracellular succinate leads to its increased uptake by macrophages, which activates a proinflammatory response and cytokine release, resulting in chronic inflammation [42]. Other investigators noted that succinate accumulation promotes an anti-inflammatory response [45,46]. For example, a recent study demonstrated that succinate induces anti-inflammatory mediators but only in lean participants with higher SUCNR1 expression [46]. This shows that nutritional status assessment is crucial in determining the effects of succinic acid accumulation. In our study, patients with inactive IBD and higher succinic acid levels had a lower BMI. It seems possible that the effect of succinic acid is dependent on body weight, including excess adipose tissue.

Our study did not show significant differences in serum cytokine levels according to disease activity. One possible explanation is that local intestinal inflammation does not affect a systemic increase in cytokine levels. This is consistent with our observation that patients with active IBD tend to have higher IL-6 mRNA expression in colon tissue with simultaneously very low serum IL-6 levels. Another reason may be the small number of participants. In our previous study on a larger group of patients, we noted higher serum TNF-α levels in patients with active IBD compared with controls, while there were no differences in IL-10, IL-17, and IL-22 levels [47]. Further studies are needed to elucidate these associations.

Considering the nutritional aspects, we noted elevated serum TNF-α levels in patients with excess body weight. Recent studies showed that obesity caused by a fatty diet increases the serum levels and intestinal expression of proinflammatory cytokines such as IL-6 and TNF-α, which are also key to the course of IBD [24]. Thus, proinflammatory markers are not only associated with disease activity, but also with a fatty diet and excessive body weight in patients with IBD. Therefore, obesity may be an important contributor to proinflammatory cytokine release and the subsequent exacerbation of colitis [48].

The main limitations of our study are the small number of participants in each study group and the incomplete dietary assessment. SCFAs are mainly formed from dietary fiber, especially the soluble fractions. However, some SCFAs and branched fatty acids, such as isovalerate and isobutyrate, can be formed by bacterial fermentation of amino acids in the large intestine [49]. Hence, it is necessary to conduct a complete dietary assessment, including a 24-h dietary interview. Due to the preliminary nature of the present study, we made a limited evaluation of the intestinal barrier markers. Therefore, we are conducting further research in this area on a larger group of participants, including a broader assessment of intestinal barrier parameters.

Recent studies showed that supplementation of probiotics and SCFAs, especially butyrate, reduce the levels of proinflammatory cytokines, such as TNF-α, IL-1β, and IL-6 [50,51,52]. This effect could be achieved through histone deacetylase inhibition and activation of G protein-coupled receptors, such as GPR41 and GPR43, located in the intestinal epithelium [51,53]. However, we did not reveal a correlation between the SCFAs and the cytokines studied. Given that the studies mentioned above were conducted on animals or cell line models, large clinical trials are needed to elucidate the exact mechanism of SCFA action and confirm this phenomenon.

## 5. Conclusions

The ongoing intestinal inflammation in IBD is affected by numerous external factors, including abnormal body weight, diet, and fiber supply. Several conclusions can be derived from our study: (1) interactions between inflammatory, intestinal barrier, and nutritional parameters should prompt a comprehensive analysis of all the factors involved; (2) the effect of the diet used, including fiber intake, may vary depending on the ongoing inflammation and baseline nutritional status of the patient; and (3) there is no single nutritional treatment for patients with IBD due to the heterogeneous characteristics of this population and the multiple external factors that affect treatment efficacy. This indicates that further clinical studies on the role of dietary pattern, fiber intake, and SCFA supplementation should account for the nutritional aspects of IBD patients. In conclusion, our study highlights the need for a holistic approach to the management of IBD patients, with concomitant personalized nutritional treatment to achieve clinical remission and mucosal healing.

## Figures and Tables

**Table 1:** Characteristics of participants and the results of standard tests in patients with inactive inflammatory bowel disease (IBD), active IBD, and controls.

Parameter	Inactive IBD *n* = 11	Active IBD *n* = 18	Controls *n* = 6	*p* Value
sex, female/male, n	2/9	6/12	4/2	-
age, years, mean ± SD (min–max)	39.8 ± 16.5 (25–71)	34.1 ± 11.7 (20–61)	43.8 ± 22.5 (20–77)	0.354
BMI kg/m^2^, mean ± SD (min–max)	21.1 ± 3.0 (16.8–26.2)	23.2 ± 5.3 (15.7–37.1)	24.2 ± 4.5 (20.5–32.9)	0.363
hemoglobin, g/dL, mean ± SD (min–max)	13.8 ± 1.8 (10.4–15.7)	12.6 ± 2.4 (7.3–16.7)	14.1 ± 2.5 (10.2–16.9)	0.148
WBC ± SD, g/L, mean ± SD (min–max)	8.0 ± 5.1 (3.7–19.1)	9.7 ± 3.0 (5.5–15.7)	7.9 ± 4.2 (4.0–10.0)	0.431
platelets, 10^3^/μL, mean ± SD (min–max)	293.1 ± 157.1 (90.5–542.0)	375.8 ± 121.7 (150.0–657.0)	224.3 ± 82.0 (160.0–343.0)	0.040 *
CRP, mg/L, median (min–max)	1.0 (1.0–57.4)	9.4 ^a^ (1.0–154.0)	1.0 ^a^ (1.0–6.0)	0.013 **
calprotectin, µg/g, median (min–max)	100.0 (0–148.0)	1541.0 ^b^ (96.0–2251.0)	<LOD ^b^ (<LOD-120.0)	0.007 **
albumin, g/L, mean ± SD (min–max)	42.8 ± 4.7 (34.1–50.3)	37.9 ± 5.9 (26.4–45.0)	44.3 ± 6.7 (32.0–49.0)	0.044 *

BMI: body mass index; CRP: C-reactive protein; IBD: inflammatory bowel disease; LOD: the limit of determination; WBC: white blood cells. * *p* value determined by the analysis of variance when comparing all three groups; however, the post-hoc Tukey test did not indicate the differences between individual groups. ** *p* value determined by the Kruskal–Wallis test when comparing all three groups. The upper-index letters “a” and “b” indicate the differing groups as determined by the post-hoc Dunn test (with *p* < 0.05 and *p* < 0.01, respectively).

**Table 2:** Clinical manifestations of patients with inactive and active IBD.

Parameter	Inactive IBD *n* = 11	Active IBD *n* = 18
Loose stools (more than 3 per day)	3 (27.3)	16 (88.9)
Abdominal pain (moderate or severe)	3 (27.3)	11 (61.1)
Anemia	2 (18.2)	8 (44.5)

Data are expressed as number (percentage) of patients.

**Table 3:** Frequency of consumption of selected food groups in patients with active IBD.

Frequency	Wholegrain Products	Dried Fruit	Raw Vegetables	Legumes
everyday	1 (5)	0	1 (5)	0
several times/week	3 (17)	0	4 (23)	0
several times/month	2 (10)	7 (39)	9 (50)	7 (39)
never	12 (78)	11 (61)	4 (22)	11 (61)

Data are expressed as number (percentage) of patients.

**Table 4:** Frequency of consumption of selected food groups in patients with inactive IBD.

Frequency	Wholegrain Products	Dried Fruits	Raw Vegetables	Legumes
everyday	1 (9)	0	1 (9)	0
several times/week	2 (18)	1 (9)	0	0
several times/month	0	2 (18)	4 (36)	4 (36)
never	8 (73)	8 (73)	6 (55)	7 (64)

Data are expressed as number (percentage) of patients.

**Table 5:** Frequency of consumption of selected food groups in controls.

Frequency	Wholegrain Products	Dried Fruit	Raw Vegetables	Legumes
everyday	1 (17)	0	1 (17)	0
several times/week	0	0	2 (34)	2 (34)
several times/month	5 (83)	2 (34)	3 (49)	1 (17)
never	0	4 (66)	0	3 (49)

Data are expressed as number (percentage) of patients.

**Table 6:** Organic acid levels in patients with inactive IBD, active IBD, and controls.

Organic Acid, µg/g	Inactive IBD *n* = 10 ^1^	Active IBD *n* = 18	Controls *n* = 5 ^1^	*p* Value
succinic	408.5 (170.4; 886.2)	208.0 (125.3; 580.9)	197.2 (91.9; 778.6)	0.466
acetic	1171.5 (906.3; 1339.6)	716.4 (552.4; 1065.1)	940.4 (770.4; 1834.9)	0.114
lactic	636.1 (44.2; 871.7)	885.3 (349.0; 2408.7)	74.5 (24.8; 1405.1)	0.313
propionic	512.1 (214.4; 920.6)	247.2 (85.9; 556.9)	322.7 (117.0; 417.2)	0.279
butyric	297.3 (35.3; 500.9)	57 (<LOD; 215.0)	118 (92.5; 227.0)	0.234
isobutyric	62.6 (<LOD; 122.3)	38.3 (<LOD; 65.4)	36.9 (32.4; 45.1)	0.518
valeric	<LOD (<LOD; 38.0)	<LOD (<LOD; <LOD)	21.3 (<LOD; 22.3)	0.503
isovaleric	62.8 (<LOD; 232.4)	<LOD (<LOD; 49.9)	77.4 (22.3–168.2)	0.036 *

Data are expressed as medians (lower and upper quartiles). LOD: the limit of determination; IBD: inflammatory bowel disease. * *p* value determined by the Kruskal–Wallis test when comparing all three groups; however, the post-hoc Dunn test did not indicate the differences between individual groups. ^1^ Data missing for one patient.

**Table 7:** Serum cytokine and zonulin levels in patients with inactive IBD, active IBD, and controls.

Parameter	Inactive IBD *n* = 10 ^1^	Active IBD *n* = 17 ^1^	Controls *n* = 5 ^1^	*p* Value
TNF-α, pg/mL	7.1 (1.2; 28.4)	5.8 (1.8; 10.8)	2.0 (0.6; 3.0)	0.187
IL-17, pg/mL	86 (70.1; 233.4)	60.9 (49.3; 251.5)	52 (40.8; 53.6)	0.061
IL-10, pg/mL	28.5 (17.9; 53.5)	20.9 (15.2; 58.1)	14.2 (13.0; 17.0)	0.079
IL-22, pg/mL	28.6 (24.2; 103.1)	30.1 (15.4; 89.7)	21.6 (19.4; 22.1)	0.315
IL-6, pg/mL	<LOD (<LOD; <LOD)	<LOD (<LOD; 4.2)	<LOD (<LOD; <LOD)	0.198
zonulin, pg/mL	2.9 (1.6; 34.4)	2 (1; 42.7)	1.9 (1.4; 2.8)	0.587

Data are expressed as medians (lower and upper quartiles). IL: interleukin; LOD: the limit of determination; IBD: inflammatory bowel disease; TNF-α: tumor necrosis factor alpha. ^1^ Data missing for one patient.

## Data Availability

The data presented in this study are available on request from the corresponding author.

## Supplementary Materials

The following supporting information can be downloaded at: https://www.mdpi.com/article/10.3390/nu14214622/s1, Figure S1: Succinic acid levels in patients on a low-fiber diet and diet with normal amount of fiber (*p* < 0.05); Figure S2: Lactic acid levels in patients on a diet with normal amount of fiber and low-fiber diet (*p* < 0.05); Figure S3: Lactic, propionic, and isovaleric acid levels in patients with diarrhea and normal bowel movement (*p* < 0.05 for all differences); Figure S4: Tumor necrosis factor alpha (TNF-α) levels in patients with a body mass index (BMI) of ≥25 kg/m^2^ and BMI of <25 kg/m^2^ (*p* < 0.05).
