# Supplementary material for: The Importance of Nutritional Aspects in the Assessment of Inflammation and Intestinal Barrier in Patients with Inflammatory Bowel Disease
Source: Nutrients. 2022 Nov 2;14(21):4622. doi: 10.3390/nu14214622 (PMC9658856; doi:10.3390/nu14214622)
Supplement: Supplementary file 1 [file nutrients-14-04622-s001.zip › nutrients-1967865-supplementary.pdf]

Supplementary material

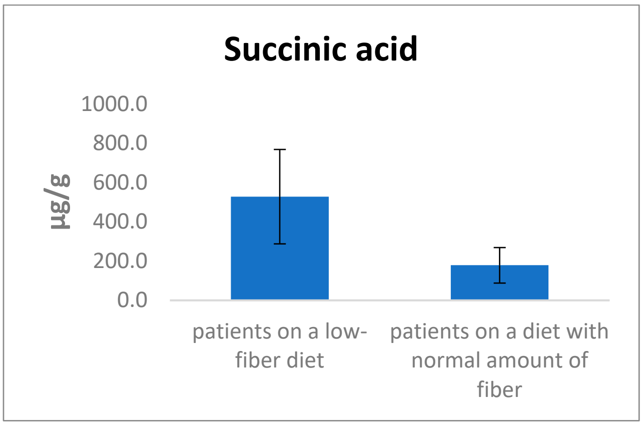

Figure S1. Succinic acid levels in patients on a low-fiber diet and diet with normal amount of fiber ($p<0.05$).

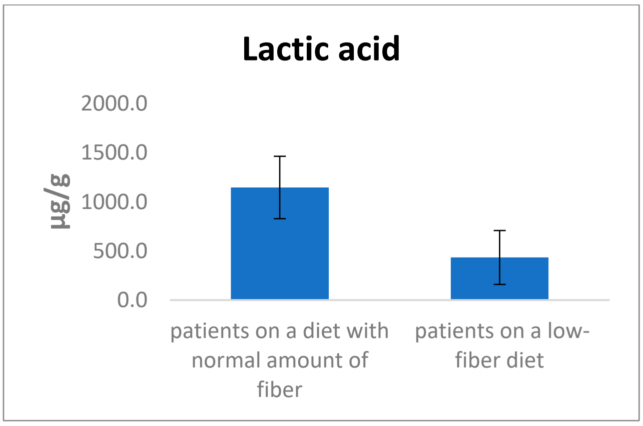

Figure S2. Lactic acid levels in patients on a diet with normal amount of fiber and low-fiber diet ($p<0.05$).

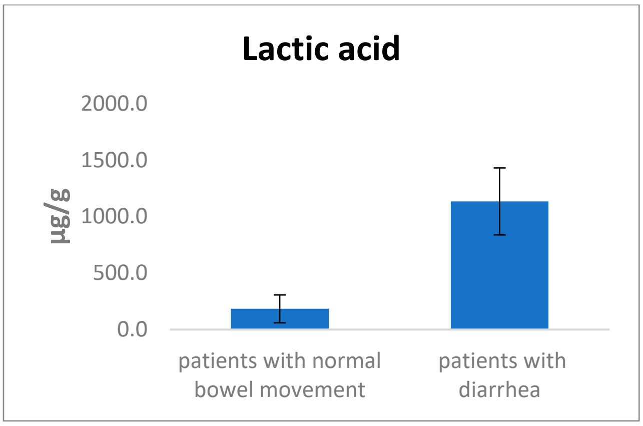

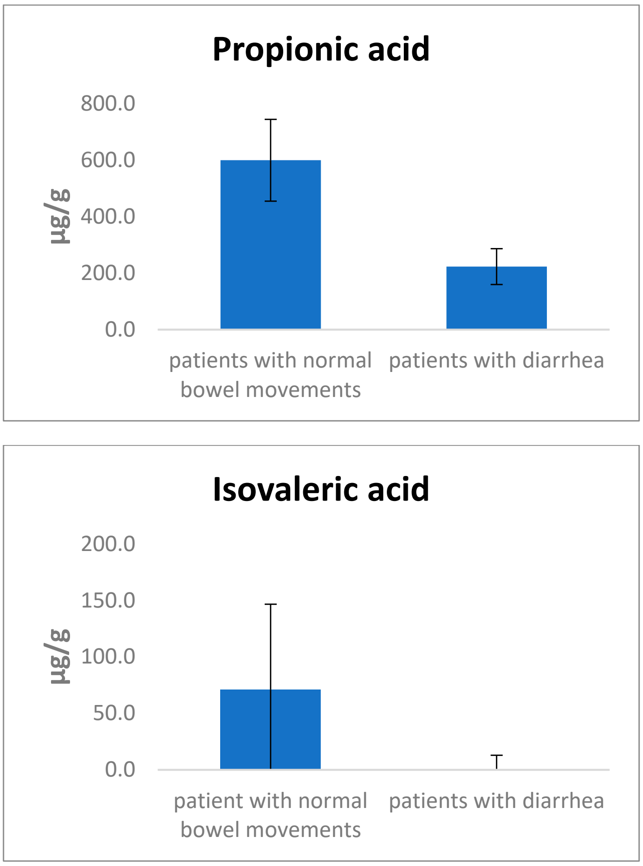

Figure S3. Lactic, propionic and isovaleric acid levels in patients with diarrhea and normal bowel movement ($p<0.05$ for all differences).

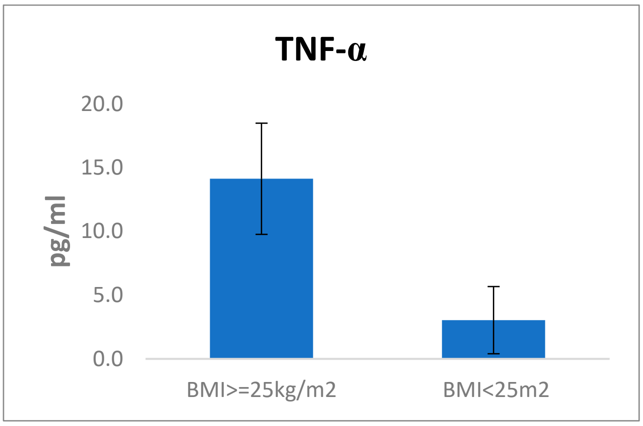

Figure S4. Tumor necrosis factor alpha (TNF-α) levels in patients with a body mass index (BMI) of ≥25 kg/m^2 and BMI of <25 kg/m^2 ($p<0.05$).
